# Observation of ordered organic capping ligands on semiconducting quantum dots via powder X-ray diffraction

**DOI:** 10.1038/s41467-021-22947-x

**Published:** 2021-05-11

**Authors:** Jason J. Calvin, Tierni M. Kaufman, Adam B. Sedlak, Michelle F. Crook, A. Paul Alivisatos

**Affiliations:** 1 Department of Chemistry, University of California, Berkeley, CA USA; 2 Material Sciences Division, Lawrence Berkeley National Laboratory, Berkeley, CA USA; 3 Kavli Energy NanoScience Institute, Berkeley, CA USA

**Keywords:** Materials chemistry, Quantum dots

## Abstract

Powder X-ray diffraction is one of the key techniques used to characterize the inorganic structure of colloidal nanocrystals. The comparatively low scattering factor of nuclei of the organic capping ligands and their propensity to be disordered has led investigators to typically consider them effectively invisible to this technique. In this report, we demonstrate that a commonly observed powder X-ray diffraction peak around $q=1.4{\AA}^{-1}$ observed in many small, colloidal quantum dots can be assigned to well-ordered aliphatic ligands bound to and capping the nanocrystals. This conclusion differs from a variety of explanations ascribed by previous sources, the majority of which propose an excess of organic material. Additionally, we demonstrate that the observed ligand peak is a sensitive probe of ligand shell ordering. Changes as a function of ligand length, geometry, and temperature can all be readily observed by X-ray diffraction and manipulated to achieve desired outcomes for the final colloidal system.

## Abstract (web summary)

The degree of ligand ordering on colloidal inorganic nanocrystal surfaces has long been a topic of interest. Here, the authors show that a well-known powder X-ray diffraction feature observed in prior works, frequently assigned to excess ligands, corresponds to bound and ordered capping ligands.

## Introduction

The degree of ligand ordering on colloidal inorganic nanocrystal surfaces has long been a topic of interest^1–8^. Capping ligands are chemically essential, providing protection against aggregation and contributing significantly to solubility^4,9,10^. Kinetic and thermodynamic properties of the nanocrystals are also greatly influenced by ligands^7–10^. There are reports of the degree of ordering led by theoretical works^2,3^ and extending to indirect probes, such as infrared spectroscopy^5,11^, small angle X-ray scattering of interparticle spacing^11,12^, and calorimetric measurements that infer entropy^5,7,8,11,12^. Such ordering is interesting fundamentally (these are well-defined finite size order–disorder transitions) and practically (influencing properties including optical, chemical, electrical, and structural). Oddly, there have been no reports of ordering between ligands detected using the most direct probes, wide angle powder X-ray diffraction and grazing incidence wide angle X-ray scattering (GIWAXS).

A variety of speculations have been proposed to explain a previously unassigned peak around $q\,=\,1.4\,{\AA}^{-1}$ often observed in routine powder X-ray diffraction characterizations of colloidal quantum dots, where $q$ is the scattering length vector. More surface-sensitive GIWAXS measurements of superlattices of lead sulfide and cadmium selenide quantum dots show a similar peak^13–15^. Explanations include unique alloys at the quantum dot surfaces^16,17^, amorphous glass slides^18,19^, oxide formation^9,10^, forbidden reflections^20,21^, and unreacted organic precursors such as indium myristate and lead oleate^22,23^. Most reports opt to ignore this peak entirely, either with a lack of explanation or failing to include it in the reported spectrum^13–15,24,25^. When aliphatic capping ligands are not used in the synthesis, this peak invariably is not present^26–29^. A more comprehensive list of articles that depict this peak alongside the given explanation is given in Supplemental Table 1. Of proposed explanations, signal from unreacted organic precursors is most compelling because the powder X-ray diffraction pattern of indium myristate, for example, has a very similar peak at $q\,=\,1.4\,{\AA}^{-1}$^22^. If the excess organic precursors are not effectively removed from the sample, this peak will be very prominent in the spectra^22,23^ and after the quantum dots are purified, this peak will decrease^23^. However, deeper analysis of powder X-ray diffraction patterns of organic precursors (Supplemental Fig. 1) and ^1^H NMR analysis of quantum dot solutions (Supplemental Fig. 2) indicate that these peaks cannot always be attributed to these explanations, especially when the quantum dots have been sufficiently purified.

Conventionally, the contribution of ordering of aliphatic ligands to powder X-ray diffraction has been considered negligible, perhaps because the scattering factors of the organic nuclei are smaller than the inorganic nuclei^30^. However, there are good reasons to believe that ordering of ligands on nanomaterial surfaces may meaningfully contribute to powder X-ray diffraction. Previous published work provides thermodynamic and optical evidence of aliphatic chains ordering on quantum dot surfaces^5,7,8^. Work published from small angle X-ray scattering (SAXS) and small angle neutron scattering (SANS) techniques on lead sulfide quantum dots in solution suggests possible ordering of ligands on the surface^6^, although this was not explicitly inferred. Additionally, ordered ligands have been reported to be observed by electron microscopy techniques^1^. Ordering of ligands has also been suggested to describe observations in melting superlattices^12,31^. The possibility that ligand ordering could be observed via quantum dot X-ray diffraction is promising considering that ordering of aliphatic chains in self-assembled monolayers has been observed in X-ray diffraction on gold surfaces^32^. Additionally, observed distances between these chains appears to coincide with the previously unassigned peak^32^. With this evidence, we elected to analyze experimental and simulated powder X-ray diffraction of different ligand treatments on indium phosphide quantum dots, where this previously unassigned peak is prominent. In this report, we show that a well-known powder X-ray diffraction feature observed in prior works frequently assigned to excess ligands^22,23^ corresponds to bound and ordered capping ligands.

## Results and discussion

### Attribution of peak to organic ligands

**Fig. 1:**
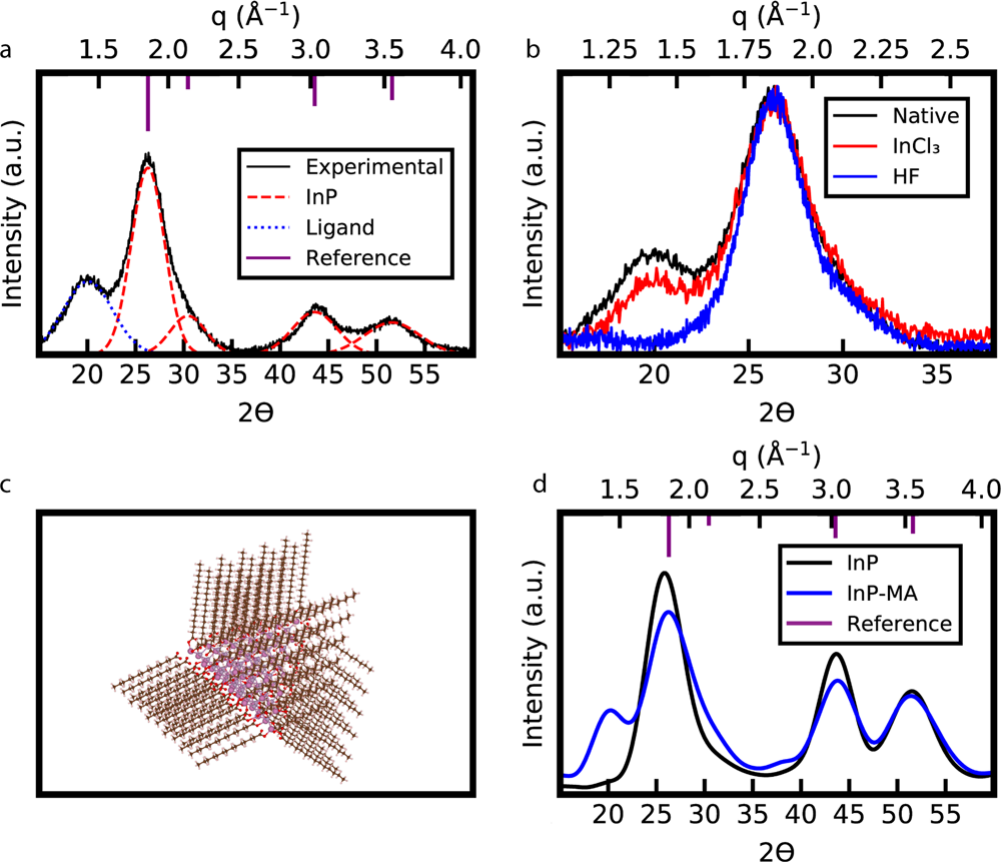
Experimental and simulated powder X-ray diffraction spectra of indium phosphide quantum dots with organic capping ligands. **a** Powder X-ray diffraction spectra with peak deconvolution of indium phosphide quantum dots capped with myristate. **b** Powder X-ray diffraction spectra of indium phosphide quantum dots with the myristate ligands partially removed by indium chloride and completely removed by hydrofluoric acid. **c** Atomic model of a tetrahedral indium phosphide quantum dot capped with myristates. **d** Simulated powder X-ray diffraction patterns of indium phosphide quantum dots with and without capping myristates (MA). Cu K-$\alpha$ $(\lambda =1.5418\; {\AA} )$ is the X-ray source for all experimental and simulated powder X-ray diffraction spectra.

A correlation between the previously unassigned peak and surface ligands was observed in experiments and simulations. The powder X-ray diffraction pattern of drop-cast indium phosphide quantum dots capped with myristate was collected and compared to the predicted peaks of zincblende indium phosphide (ICSD 1600543) (Fig. 1a). To illustrate the presence of the ligand peak, Gaussian peaks were used as a crude approximation to deconvolute the powder X-ray diffraction, although powder X-ray diffraction peaks do not strictly have Gaussian or Lorentzian line shapes^33^. In addition to expected diffraction peaks, the previously unassigned peak is observed at approximately $q\,=\,1.4\,{\AA}^{-1}$. Different treatments to the indium phosphide quantum dots can modulate this peak (Fig. 1b). Treatment with indium chloride (InCl_3_) reduces the relative intensity of the ligand peak to the (111) diffraction peak, while treatment with hydrofluoric acid (HF) removes the ligand peak entirely. InCl_3_ has been shown to partially displace indium myristate from indium phosphide quantum dot surfaces in a Z-type ligand exchange^8^, while HF protonates the carboxylates on the quantum dot surface and replaces them with fluorine^34^. From these measurements, we postulated that ordered myristates on the quantum dot surfaces were the source of these diffraction peaks. To further investigate this possibility, powder X-ray diffraction simulations were performed on an indium terminated charge balanced atomic structure of a tetrahedral indium phosphide quantum dot. The quantum dot was capped with ordered myristate ligands at ligand densities that have been observed for indium phosphide quantum dots (Fig. 1c)^7,8,35^. While the synthesized indium phosphide quantum dots are likely not perfectly faceted^8^, and the co-ordination of the capping carboxylates to the surface indiums is not precisely defined due to variability in their coordination motifs^36^, this provides a model system to evaluate the hypothesis that this peak comes from ordered capping aliphatic chains. The simulated powder X-ray diffraction pattern of the quantum dot with ligands contained a peak at $q\,=\,1.4\,{\AA}^{-1}$, while the powder X-ray diffraction pattern of the quantum dot without capping ligands lacked this previously unassigned peak (Fig. 1d). The ability to reproduce this peak in the simulations provides further support that this feature originates from ordered myristates on the quantum dot surfaces. Not only is this peak seen in indium phosphide, but such features have also been observed in a variety of other colloidal nanocrystals, such as cadmium selenide quantum dots capped with oleates (Supplemental Figs. 3 and 4).

### Size dependence of identified ligand peak

**Fig. 2:**
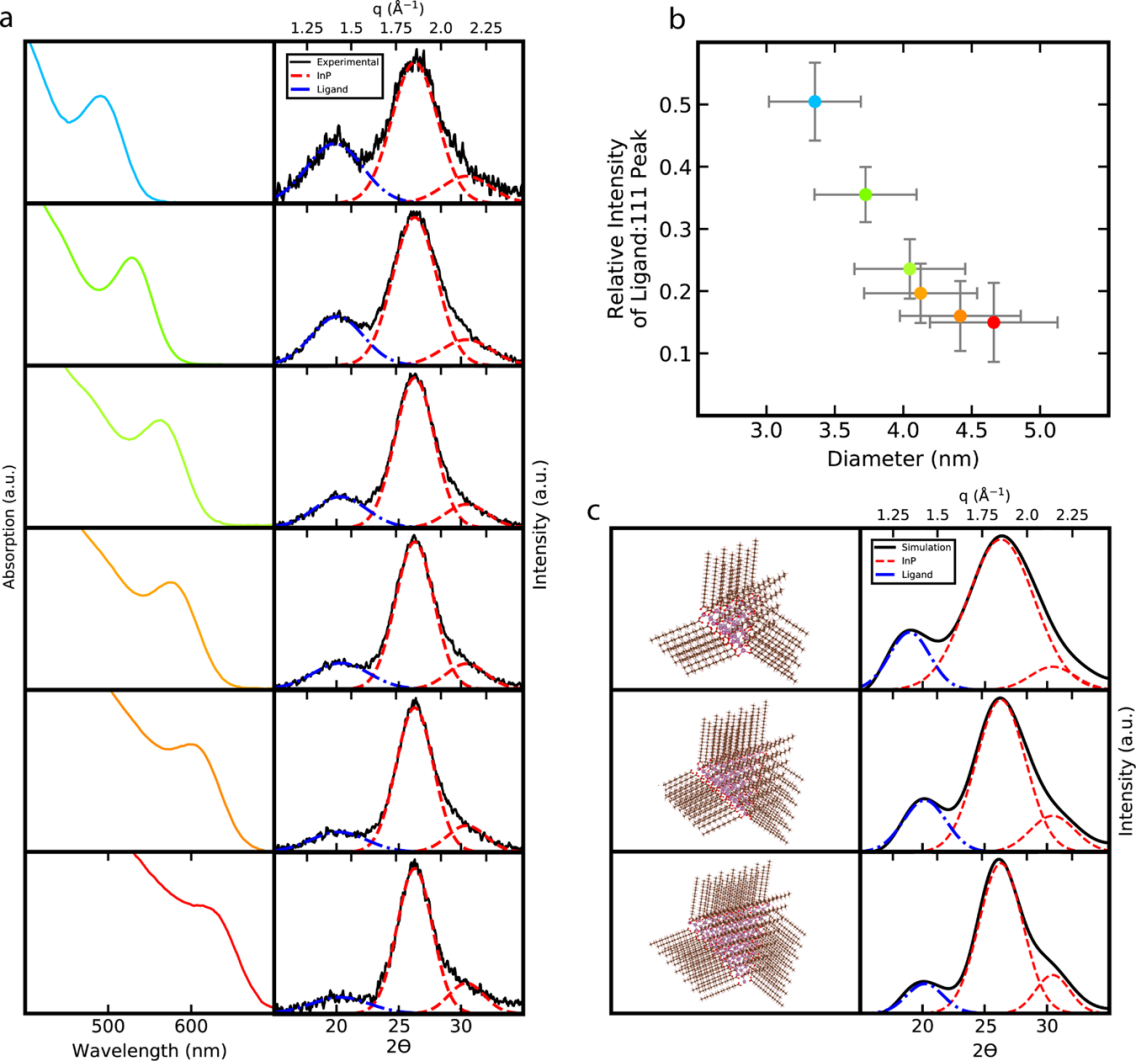
Experimental and simulated powder X-ray diffraction spectra of a size series of indium phosphide quantum dots determined by absorption spectra. **a** Absorption spectra of indium phosphide quantum dots indicating the size of the quantum dots coupled with the powder X-ray diffraction spectra. **b** Relative intensity of the ligand peak to the (111) diffraction peak of indium phosphide as a function of quantum dot size determined by powder X-ray diffraction peak deconvolution. Error in relative intensity was determined by propagation of the standard error in counts per second from the detector and error in nanocrystal size was taken at 10% from reported errors in sizing calculation functions determined by UV–Vis spectroscopy (see “Methods” section). **c** Atomic models of a size series of indium phosphide quantum dots and the corresponding simulated powder X-ray diffraction patterns. Cu K-$\alpha$ $(\lambda =1.5418\; {\AA})$ is the X-ray source for all experimental and simulated powder X-ray diffraction spectra.

A series of experiments revealed that as quantum dot size increases, the relative intensity of the organic ligand peak to the inorganic (111) diffraction peak decreases. Larger quantum dots should have a smaller proportion of organic material represented in their powder X-ray diffraction, meaning this ligand peak should effectively disappear from the powder X-ray diffraction pattern once the nanocrystal size becomes sufficiently large (Supplemental Fig. 4). To investigate this implication, indium phosphide quantum dots were synthesized with diameters varying between 3.4 and 4.6 nm, as determined by their absorption spectra, and corresponding powder X-ray diffraction patterns were collected (Fig. 2a). As predicted, with increasing quantum dot size, the relative intensity of the ligand peak decreased in both experimental (Fig. 2b), and simulated powder X-ray diffraction patterns (Fig. 2c).

### Ligand exchange and peak modulation

**Fig. 3:**
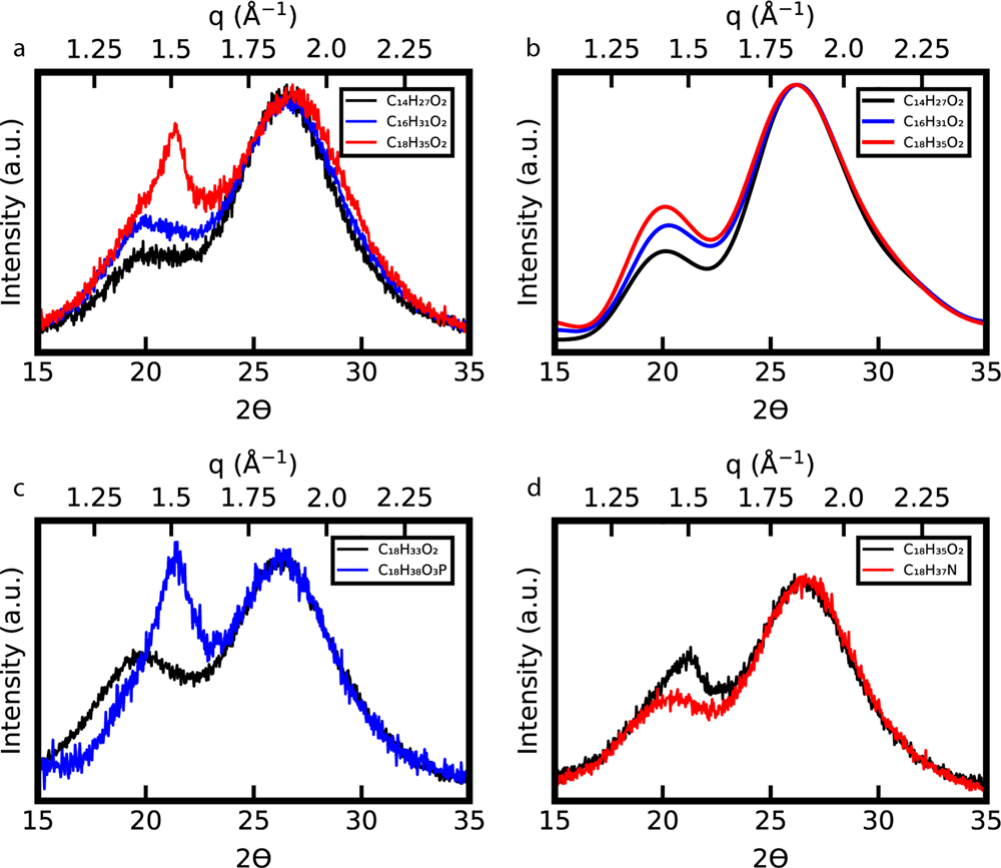
Experimental and simulated powder X-ray diffraction spectra of indium phosphide quantum dots capped with carboxylates of different lengths. **a** Experimental powder X-ray diffraction spectra of indium phosphide quantum dots capped with myristate (C_14_H_27_O_2_), palmitate (C_16_H_31_O_2_), and stearate (C_18_H_35_O_2_) ligands. **b** Simulated powder X-ray diffraction spectra of indium phosphide quantum dots capped with myristate, palmitate, and stearate ligands. **c** Experimental powder X-ray diffraction spectra of indium phosphide quantum dots initially capped with oleate (C_18_H_33_O_2_) and after ligand exchange with octadecylphosphonate (C_18_H_38_O_3_P). **d** Experimental powder X-ray diffraction spectra of indium phosphide quantum dots initially capped with stearate and after ligand exchange with oleylamine (C_18_H_37_N). Note that the powder X-ray diffraction pattern of stearate (C_18_H_35_O_2_) capped indium phosphide quantum dots in **d** differs from the one in panel **a** as the quantum dots are larger and from a separate synthesis. Cu K-$\alpha$ $(\lambda \,=\,1.5418\,{\AA})$ is the X-ray source for all experimental and simulated powder X-ray diffraction spectra.

If this peak originates from packing of organic chains on the nanocrystal surface, changing the nature of the ligand on the surface should affect the shape of the previously unassigned powder X-ray diffraction peak. Powder X-ray diffraction patterns of indium phosphide quantum dots capped with myristate (C_14_H_27_O_2_), palmitate (C_16_H_31_O_2_), and stearate (C_18_H_35_O_2_) ligands were collected (Fig. 3a). As the length of the carbon chain increases, the relative intensity of the ligand peak increases due to a greater number of carbons atoms contributing to the diffraction signal. Powder X-ray diffraction simulations predict this same trend (Fig. 3b). These results suggest that with very short ligands, the ligand peak may visibly disappear and not be observed unless purposely inspected for through peak deconvolution techniques.

Interestingly, the indium phosphide quantum dots capped with stearate have a more intense and sharper peak than those capped with myristate and palmitate. Previous work on carboxylate ligand exchanges suggests that internal rotation restriction of stearate is more severe than that of palmitate and myristate ligands^7^. Stearate ligands also have stronger interligand Van der Waals interactions than palmitate and myristate^7^. Furthermore, the difference in these interactions between palmitate and stearate ligands is much larger than between myristate and palmitate ligands, despite both cases having a two carbon difference in chain length^7^. This suggests that there are fundamental changes to the ligand shell structure. Shifting of the ligand powder X-ray diffraction peak to larger $q$ values indicates a decrease in distance between chains, consistent with stronger van der Waals interactions and restrictions on internal rotations. Sharpening of the peak as well as greater intensity may also indicate possible ordering between ligands attached to different nanocrystals. Additionally, this sharp peak in the quantum dot powder X-ray diffraction pattern is similar in location and shape to that of recrystallized indium myristate; however, as stated it is unlikely to be the cause of this previously unassigned peak (Supplemental Fig. 1b).

Balan et al. predict a “melting” phase transition in the ligand shell above room temperature for colloidal quantum dots capped solely with octadecylphosphonate (C_18_H_38_O_3_P)^5^. We propose that the change in the observed stearate powder X-ray diffraction pattern is due to myristate and palmitate ligand shells already having undergone this phase transition at room temperature, although they still retain some ordering. For indium phosphide quantum dots, when oleate (C_18_H_33_O_2_) capping ligands (a ligand containing a *cis*-alkene that hinders ordering and interligand interactions) are exchanged for octadecylphosphonate ligands (a long, straight chained ligand) the resulting powder X-ray diffraction has the same sharpness observed in the stearate capped particle spectrum (Fig. 3c). Similarly, when indium phosphide nanocrystals capped with stearate have their ligands partially exchanged for oleylamine (C_18_H_37_N), another ligand containing a *cis*-alkene, the peak broadens and shifts to smaller $q$ values (Fig. 3d).

Sandhyarani et al. provides further experimental evidence of this phenomenon through their study of superlattices of silver nanoparticles capped with alkylthiol ligands, particularly those capped with octadecanethiol (C_18_H_37_S)^12^. Differential scanning calorimetry of the superlattices revealed two peaks in the heating curve: a smaller peak around 325 K and a larger peak around 405 K. The smaller peak was attributed to the transition to a liquid phase that still retained alkyl chain order, while the larger peak was attributed to the transition to a liquid phase, where alkyl chain order was no longer present^5^. Ordering of the alkyl chains in these superlattices was also observed by Fourier transform infrared spectroscopy^11^. We suggest that myristate and palmitate capped particles are in the first liquid phase that still retains order, while stearate capped particles are in the more crystalline phase. Interestingly, in the powder X-ray diffraction pattern reported by Sandhyarani et al., a peak attributed to the superlattices around 20° $2\theta$ is present that is wider than the other superlattice peaks. We suspect this feature is actually from the ordering of ligands on the nanoparticle surfaces^12^. Furthermore, differential scanning calorimetry thermograms on indium phosphide magic clusters capped with myristate alongside ^13^C NMR provide evidence of a small phase transition of the ligand shell at 339 K^22^. Similar differential scanning calorimetry measurements have reported a phase transition attributed to oleate ligands melting and freezing on the surface of lead sulfide nanocrystals in superlattices in the range of 240–270 K. These results are all consistent with our hypotheses about the nature of the ligand shell and its dependence on the types of aliphatic ligands and the temperature, where *cis*-alkenes cause a lowering of the phase transition and have weaker intermolecular interactions and less ordering while longer, straight-chain alkanes are more ordered^5,7,8^.

### Temperature dependence of ligand peak

**Fig. 4:**
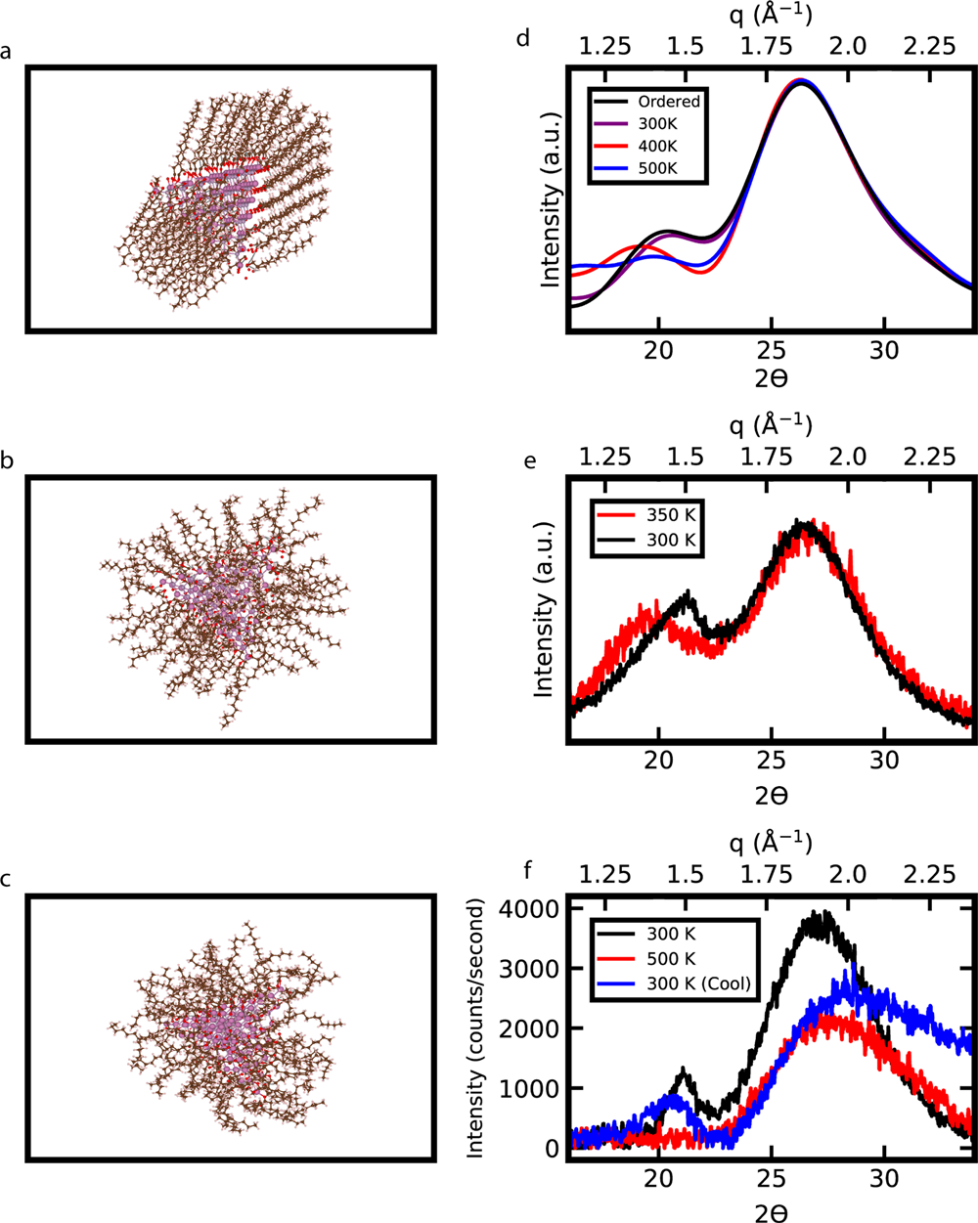
Atomic models and simulated powder X-ray diffraction spectra of indium phosphide quantum dots with ligand ordering simulated through molecular dynamics compared with experimental data. **a** Atomic model of an indium phosphide quantum dot capped with myristates after molecular dynamics simulations at 300 K. **b** Atomic model of an indium phosphide quantum dot capped with myristates after molecular dynamics simulations at 400 K. **c** Atomic model of an indium phosphide quantum dot capped with myristates after molecular dynamics simulations at 500 K. **d** Simulated powder X-ray diffraction spectra of indium phosphide quantum dots before and after molecular dynamics simulations. **e** Experimental powder X-ray diffraction spectra of indium phosphide quantum dots capped with stearate depicting the peak shifting and decreasing in intensity at an increased temperature. **f** Experimental powder X-ray diffraction spectra of indium phosphide quantum dots capped with stearate showing the disappearance of the ligand peak at 500 K followed by its reappearance when cooled back to 300 K. Note that patterns depicted in **e** and **f** were measured on different instruments. The broadening of the (111) diffraction peak in the heated and cooled spectra is evidence that the sample may have slightly degraded due to sustained high temperatures. Cu K-$\alpha$ $(\lambda =1.5418\; {\AA})$ is the X-ray source for all experimental and simulated powder X-ray diffraction spectra.

To further investigate our hypothesis concerning the ordering of the ligand shell, we performed temperature dependent molecular dynamics simulations on our atomic model and experimental temperature dependent powder X-ray diffraction on stearate capped indium phosphide quantum dots. Previous powder X-ray diffraction simulations were performed on atomic systems with perfectly aligned ligands on the nanocrystal, but this is not a strictly accurate representation, particularly of indium phosphide quantum dots capped with shorter chain ligands. After performing molecular dynamics simulations at 300, 400, and 500 K (Fig. 4a–c), we observed that ligands are still fairly ordered at 300 K, forming clumps of ligands that appear to be slightly less ordered at the edges. The ligands became more disordered as temperature increased, as seen from previous molecular dynamics simulations of ligands on the nanoparticle surfaces^2,3^. The simulated powder X-ray diffraction patterns show that as temperature increases, the peak broadens and shifts to smaller $q$ values, indicating wider ligand spacing that is attributed to increased disorder and weaker interactions (Fig. 4d). Experimental powder X-ray diffraction patterns reveal that at room temperature the stearate ligand peak is sharp and located at larger $q$ values; however, upon raising the temperature in the powder X-ray diffraction sample holder, from 300 K to the 350 K, the peak shifts to smaller $q$ values and broadens (Fig. 4e). This is consistent with the behavior observed in the molecular dynamics simulations and analysis in Fig. 3. When the sample is heated from 300 to 500 K the peak completely disappears, but when it is cooled back to 300 K the peak reappears (Fig. 4f). These results confirm the reversibility of the ligand shell phase transition previously observed by Sandhyarani et al. with silver nanocrystal superlattices^12^.

### Implications for nanocrystal characterization

From this suite of measurements, we conclude that the previously unassigned peak often observed at $q\,=\,1.4\,{\AA}^{-1}$ originates from ordered aliphatic ligands on the quantum dot surfaces. Not only does this conclusion assist in clarifying commonly observed results in powder X-ray diffraction and GIWAXS patterns of small nanoparticles capped by long carbon chains, but also has much broader implications. For example, structural information about the ligands on small quantum dot surfaces can be deduced from a relatively routine characterization method, powder X-ray diffraction. We encourage future characterization of nanocrystals to consider this peak. Although ligand peaks in larger nanocrystals may be significantly dwarfed by their inorganic counterparts, they should still be detectable through careful measurement and peak deconvolution (Supplemental Fig. 4).

More broadly, measurement and manipulation of surface ligand ordering can have significant impact on many properties of colloidal quantum dots. Highly ordered ligands on colloidal quantum dot surfaces can have detrimental effects on optical properties^5,37–39^. Disordered surface ligands also increase the solubility of nanoparticles, and understanding the dynamics between ligand type and ligand length on their ordering could enable more precise tuning of solubility^4^. Additionally, ligands have been shown to cause tensile stress to the nanoparticle surfaces, which is likely enhanced by more crystalline phase ligands^40,41^. Powder X-ray diffraction measurements could also potentially tease out the effects of ligand exchanges and mixed ligand shells. For example, evidence has been shown that mixed ligand shells may not randomly distribute their ligands, but rather form patchy surfaces^7,8,42^. In certain cases, powder X-ray diffraction may be used to observe these effects.

The field most likely to benefit from this work involves the formation of nanocrystal superlattices. As observed by Sandhyarani et al., the melting behavior of superlattices depends on these types of ordering interactions^12,31^. The use of GIWAXS to identify and evaluate this feature alongside SAXS, and grazing incidence small angle X-ray scattering (GISAXS) to characterize and analyze superlattices should yield further insight into the superlattice structures^13–15,43^. Altering the phase of the ligands could potentially manipulate the ligand ordering behavior to change distances between nanocrystals or modify superlattice assembly^43–46^. We anticipate that these techniques can assist in optimizing future superlattices through employment of ligands with appropriate crystallization properties, and maintaining temperatures during superlattice formation that encourage certain ligand shell behavior. In general, these measurements can help develop design rules for ligands to engineer the preferred type of organic shell. Optimization of nanostructures requires a thorough understanding of their properties, particularly at their surfaces, and powder X-ray diffraction provides one additional tool for characterizing ligands on nanocrystal surfaces. This simple, yet effective method has revealed far more about the ligand shell structure than previously thought possible.

## Methods

### Chemicals

Indium acetate (99.99%), myristic acid (≥99%), indium chloride (99.999%), and hexanes (>99%) were purchased from Sigma-Aldrich and stored in an argon glovebox. Tetrahydrofuran (≥99.9%, Sigma-Aldrich) was distilled from a solvent still immediately before being stored in an argon glovebox. Tris(trimethylsilyl)phosphine (98%, Strem Chemicals) and acetone (99.8%, Acros Organics) were also stored in an argon glovebox. Palmitic acid (99%), oleic acid (90%), 1-octadecene (90%), oleylamine (70%), and hydrofluoric acid (99.99%, 48 wt% in H_2_O) were purchased from Sigma-Aldrich and used as received. Stearic acid (98%, Alfa Aesar), octadecylphosphonic acid (>99%, PCI Synthesis), dimethyl sulfoxide (99.9% Fisher Scientific), argon (99.999%, Airgas), and hydrogen (5% in argon, Airgas) were used as received.

### Indium phosphide quantum dot synthesis

This synthesis and characterization of the indium phosphide quantum dots for comparison of carboxylate lengths has previously been reported^7,8^. For the synthesis, 0.350 g (1.20 mmol) of indium acetate with 3.60 mmol of the targeted carboxylic acid (0.822 g of myristic acid, 0.923 g of palmitic acid, 1.024 g of stearic acid, and 1.017 g of oleic acid) were combined with 10 mL of 1-octadecene in an oven-dried, 50 mL three neck round-bottom flask with a Teflon coated stir bar. After an air condenser column, septa, and thermocouple adapter were attached to the flask and all glass joints were greased with Apiezon H grease, the flask was heated using a heating mantle and evacuated via Schlenk line for 1 h at 110 °C at a pressure of 60 mTorr, resulting in a clear solution. The system was filled with ultra-high purity argon and the temperature was raised to 130 °C. In an argon glovebox, 1.578 g of 1-octadecene that had previously been heated and degassed for an hour at 110 °C at 60 mTorr was combined with 0.152 g of tris(trimethylsilyl)phosphine (0.607 mmol) and loaded into a syringe. This solution was then swiftly injected into the flask and temperature was maintained for 2 min, resulting in a yellow solution. The temperature was then raised at a rate of 20 °C/min to 230 °C and maintained for 5 min for the quantum dots capped with myristic and palmitic acid and 15 min for the quantum dots capped with stearic and oleic acid. This longer growth time for the quantum dot chains capped with longer ligands was intended in an attempt to make particles of similar sizes with different capping carboxylates. The solution turned a dark red over the growth period, and the flask was then rapidly cooled under a stream of nitrogen and hexanes. It was then cannulated to be purified in an argon glovebox. To the solution, 36 mL of acetone was added to precipitate the quantum dots, and the solution was centrifuged at 5702 RCF. The supernatant was discarded, and 4 mL of tetrahydrofuran was added to suspend the quantum dots. Note that hexanes was used instead of tetrahydrofuran for the resuspensions of: (1) the sample of myristic acid capped quantum dots to be treated with hydrofluoric acid and (2) the sample of stearic acid capped quantum dots to later undergo a ligand exchange with oleylamine, hexanes was used instead of tetrahydrofuran for resuspensions. An additional 12 mL of acetone was added, and resulting precipitate was centrifuged again and resuspended in an additional 4 mL of tetrahydrofuran.

This cleaning process was repeated an additional time for the stearic acid capped quantum dots for the ligand exchange with oleylamine, and after resuspension in hexanes, 8 mL of acetone was added. The solution became cloudy, forming a white precipitate. This solution was centrifuged, the supernatant was retained, and an additional 2 mL of acetone was added to the solution, forming another cloudy suspension. After centrifugation, the resulting red pellet was resuspended in 2 mL of hexanes.

### Indium phosphide-H_2_ quantum dot synthesis

The preparation of the size series of indium phosphide quantum dots was nearly identical to that described for the indium phosphide quantum dots above with a few differences. For the synthesis, 0.350 g (1.20 mmol) of indium acetate with 0.822 g of myristic acid (3.60 mmol) were combined with 10 mL of 1-octadecene in an oven-dried, 50 mL three neck round-bottom flask with a Teflon-coated stir bar. After an air condenser column, septa, and thermocouple adapter were attached to the flask and all glass joints greased with Apiezon H grease, the flask was evacuated via Schlenk line. The temperature of the flask was raised slowly to 70 °C, after which the vacuum line was closed. The solution was slowly heated to 80 °C, and the line to the vacuum was then opened slightly. The solution was then slowly heated to 120 °C, and all solids disappeared, resulting in a clear solution. Maximum temperature reached by the solution tended to be 130 °C. The temperature was lowered to 90 °C, after which the vacuum line was fully opened, reaching a pressure below 60 mTorr. The system was flushed with 5% hydrogen in argon gas and placed back under vacuum, and this process was repeated three times in intervals of 10 min. In total, the degassing process lasted 1 h. The system was filled with 5% hydrogen in argon and the temperature was raised to 130 °C. In an argon glovebox, 1.578 g of 1-octadecene that had previously been heated and degassed for an hour at 110 °C at 60 mTorr was combined with 0.152 g of tris(trimethylsilyl)phosphine (0.607 mmol) and loaded into a syringe. This solution was then swiftly injected into the flask, and temperature was maintained for 2 min, resulting in a yellow solution. The temperature was then raised to 190 °C at a rate of 20 °C/min and maintained at this temperature between 1 and 45 min, with longer reaction times yielding larger particles. The solution turned a deep red to completely black depending on the length of the growth period, and then the flask was then rapidly cooled under a stream of nitrogen and hexanes. After cooling the solution was cannulated to be purified in an argon glovebox. To the solution, 36 mL of acetone was added to precipitate the quantum dots, and the solution was centrifuged at 5702 RCF. The supernatant was discarded, and 4 mL of hexanes was added to suspend the quantum dots. An additional 12 mL of acetone was added, and resulting precipitate was centrifuged again and resuspended in an additional 4 mL of hexanes. This process was repeated one more time, after which acetone was added dropwise to the solution until a precipitate appeared and persisted. After centrifugation, a white solid appeared, and the supernatant was retained. Additional acetone was added until a total of 8 mL of acetone had been added, causing additional precipitation. After centrifugation, a red to black pellet was obtained leaving behind a yellow solution which was discarded. The pellet was then suspended in 4 mL of hexanes.

### Indium chloride ligand exchange

The treatment of indium phosphide quantum dots with indium chloride has previously been reported^8^. The concentration of indium phosphide quantum dots, synthesized in absence of a hydrogen atmosphere and capped with myristic acid, was calculated from UV–Vis measurements. Then, 1.2 mL of a 32 µM in indium phosphide quantum dot solution was prepared using tetrahydrofuran as the solvent. Subsequently 250 µL of 93 mM concentrated indium chloride in tetrahydrofuran was added to the solution and stirred. The solution was then washed with acetone in a 3:1 ratio to precipitate the quantum dots, after which the solution was centrifuged at 5702 RCF for 5 min and the supernatant was discarded. The quantum dots were then resuspended in 1 mL of tetrahydrofuran, and the process was repeated again to remove residual indium myristate or indium chloride that remained in solution.

### Octadecylphosphonic acid ligand exchange

The ligand exchange of octadecylphosphonic acid with myristate capped indium phosphide quantum dots has previously been reported^7^. For exchange, 1.2 mL of 8 µM indium phosphide quantum dots capped with oleic acid synthesized in absence of a hydrogen atmosphere was titrated with 230 µL of a 16 mM solution of octadecylphosphonic acid. After treatment the solution was washed with acetone in a 3:1 ratio to precipitate the quantum dots, after which the solution was centrifuged at 5702 RCF for 5 min and the supernatant was discarded. The quantum dots were then resuspended in 1 mL of tetrahydrofuran and the process of adding acetone and resuspending in tetrahydrofuran was repeated.

### Oleylamine ligand exchange

For the exchange, 1 mL of the stock solution of indium phosphide quantum dots capped with stearic acid synthesized in absence of a hydrogen atmosphere was treated with 20 µL of oleylamine that had been degassed at 90 °C for 1 h. The quantum dots were then precipitated with 3 mL of acetone, and then resuspended in 1 mL of hexanes. An additional 20 µL of oleylamine was added and then the washing process was repeated three more times without the addition of any more oleylamine.

### Hydrofluoric acid treatment

Treatment with hydrofluoric acid was adapted from the procedure by Kim et al.^25,34^. A stock solution of myristic acid capped quantum dots not prepared with a hydrogen atmosphere was diluted to 15 mL in hexanes to make a 20 µM solution. In a Teflon vessel, this solution was added with 15 mL of dimethyl sulfoxide and 52 µL of hydrofluoric acid. The vessel was sealed with a Teflon cap and a steel pressure vessel and shaken thoroughly for 1 min. The vessel was then placed in a boiling water bath for 30 min, after which it was allowed to sit to cool to room temperature. The vessel was opened and the contents transferred to a separatory funnel, where the solution separated into two layers, a reddish layer on the bottom and a relatively clear layer on top. Additionally, a red mass formed at the interface of the two layers, which was attributed to be myristic acid contaminated with residual indium phosphide quantum dots. The lowest layer was drained and saved while the upper layer was discarded. The lower layer was placed back into the separatory funnel and 15 mL of hexanes was added. The funnel was capped and shaken, and the layers were allowed to separate. After this the process of washing with hexanes was repeated an additional four times.

### Optical characterization

Stock solutions of quantum dots were diluted in tetrahydrofuran by adding 30 µL of the stock solution to 3 mL of tetrahydrofuran or hexanes (depending on the solvent of the stock solution) in a 1 cm path length quartz cuvette prepared in an argon glovebox. The cuvettes were sealed, and absorption measurements were taken on a Shizmadu UV-3600 spectrometer with a slit width of 1.0 nm and a scanning speed of 350 nm/min with a resolution of 1.0 nm.

Size and concentration measurements of the quantum dots were performed using absorption measurements. Using the estimated position of the first exciton peak ($\lambda$) and the size correlation function^47^, the diameter (*D*) of the particles was calculated.

1 $$D=	\left(-3.77074\,\times\,{10}^{-12}\right){\lambda }^{5}+\left(1.0262\,\times\,{10}^{-8}\right){\lambda }^{4}-\left(1.0781\,\times\,{10}^{-5}\right){\lambda }^{3}\\ 	+\left(5.4500\,\times\,{10}^{-3}\right){\lambda }^{2}-\left(1.3122\right)\lambda +119.9$$

After the diameter was calculated, the molar extinction coefficient correlation function^47^ was used to calculate the molar extinction coefficient (*ε*).

2 $$\varepsilon\,=\,3046.1{\left(D\right)}^{3}-76532{\left(D\right)}^{2}+\left(5.5137\,\times\,{10}^{5}\right)\left(D\right)-\left(8.9839\,\times\,{10}^{5}\right),$$

Using the Beer Lambert law, the concentration of the quantum dot solutions was then calculated, where *A* is the absorbance, *ε* is the molar extinction coefficient in L mol^−1^ cm^−1^, *b* is the pathlength in cm, and *C* is the concentration of the solution in mol L^−1^.

3 $$A=\varepsilon {bC}$$

Estimated standard error in diameters and concentration is 10%.

### Powder X-ray diffraction

Quantum dot solutions were drop-cast onto a single crystal silicon wafer from high vapor point solvents such as tetrahydrofuran and hexanes. They were allowed to dry quickly to prevent superlattice formation of the nanocrystal powder and instead produce randomly oriented nanocrystals. Measurements were done on a Bruker D2 Phaser instrument and samples were irradiated with copper K-alpha X-rays with a wavelength of 1.5418 Å. Spectra were collected at angles between 10° and 70° $2\theta$ under φ angular rotation of 72°/s. Spectra were baselined using a rubber-band baseline construction using a spectrum of the blank silicon wafer as a guide.

### Temperature dependent powder X-ray diffraction

Quantum dot solutions were dried into a thick paste in the sample holder. The X-ray diffraction patterns of the samples were collected using a Rigaku MiniFlex 6G X-ray diffractometer with a copper K-alpha radiation (*λ* = 1.5418 Å). The voltage and current were 40 kV and 15 mA respectively. The scanning rate was 5 °C/min in the range of 2*θ* from 10° – 40°.

### Powder X-ray diffraction simulations

Powder X-ray diffraction patterns were simulated on structures built in Gaussian 16 and manipulated in Avogadro. Tetrahedrons of indium phosphide were constructed with a ligand density of 8 ligands/nm^2^, approximately consistent with ligand density measurements previously reported for nanocrystals synthesized in the manner described herein. Intensities ($I$) as a function of the scattering length vector ($q$) in units of Å^−1^ were calculated using the Debye formula

4 $$I\left(q\right)=\mathop{\sum} _{{\rm{i}}}\mathop{\sum} _{{\rm{j}}}{f}_{{\rm{i}}}\left(q\right){f}_{{\rm{j}}}\left(q\right)\frac{{{\sin }}\left(q{r}_{{{\rm{ij}}}}\right)}{q{r}_{{{\rm{ij}}}}},$$

where the sums are over all of the atoms, $f$ is the angle-dependent scattering factor calculated by Hartree-Fock wavefunctions by Cromer and Mann, and $r$ is the distance between atoms in Å^30,35^. The scattering length vector was converted to $2\theta$ using the formula

5 $$2\theta\,=\,2\,{{\arcsin }}\left(\frac{q\lambda }{4\pi }\right),$$

where $\lambda$ is the wavelength of incident X-rays (1.541 Å) for plotting against experimental data.

### Molecular dynamics simulations

Molecular dynamics simulations were performed in Maestro using Desmond. Structures using for powder X-ray diffraction simulations were imported and indium and phosphorus atoms were removed. Carboxylate were replaced by methyl groups. Structures were then prepared via the System Builder using the OPLS2005 force field. For the simulation, terminal methyl carbons near the interior of the structure were fixed with a force constant of 100 kcal mol^−1^ Å^−1^. Simulation temperature was selected, and the simulation was run for 1.2 ns with a recording interval of 4.8 picoseconds. After simulation, the resulting structure was exported into Avogadro, the carboxylates were reinstated as well as the indium phosphide core for powder X-ray diffraction simulation.

## Supplementary information

Supplementary Information

## Data Availability

The datasets generated during and/or analyzed during the current study are available from the corresponding author on reasonable request.
