# Supplementary material for: Observation of ordered organic capping ligands on semiconducting quantum dots via powder X-ray diffraction
Source: Nat Commun. 2021 May 11;12:2663. doi: 10.1038/s41467-021-22947-x (PMC8113276; doi:10.1038/s41467-021-22947-x)
Supplement: Supplementary file 1 — Supplementary Information [file 41467_2021_22947_MOESM1_ESM.pdf]

Supplemental Information for Observation of Ordered Organic Capping Ligands on Semiconducting Quantum Dots via Powder X-ray Diffraction

Jason J. Calvin[1,2], Tierni M. Kaufman[1], Adam B. Sedlak[1], Michelle F. Crook[1,2], and A. Paul Alivisatos[1,2,3]*

[1]Department of Chemistry, University of California, Berkeley, California 94720, United States. [2]Material Sciences Division, Lawrence Berkeley National Laboratory, Berkeley, California 94720, United States. [3]Kavli Energy NanoScience Institute, Berkeley, California 94720, United States. *email: paul.alivisatos@berkeley.edu

Supplementary Discussion

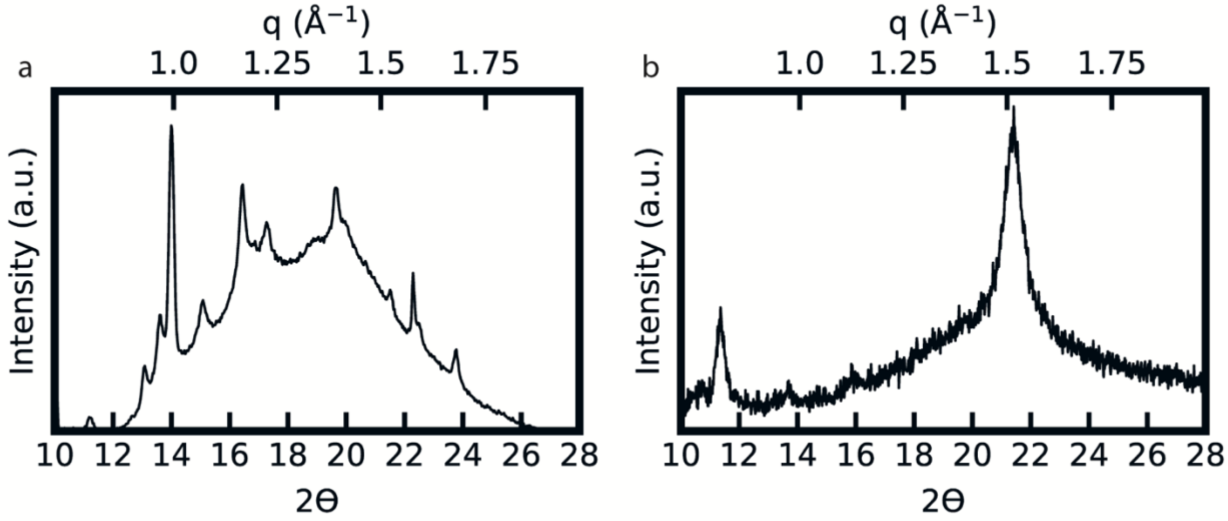

Supplemental Figure 1. **Powder X-ray diffraction spectra of synthesized indium myristate.** **a**, Drop cast from solution. **b**, Recrystallized from acetone. Cu K-α ($\lambda = 1.5418$ Å) is the X-ray source for the powder X-ray diffraction spectra.

One of the most common explanations for the ligand peak is unreacted organic precursor.[1, 2] Based on the spectra of drop cast and recrystallized indium myristate around $q = 1.4$ Å^{-1}, where q is the scattering length vector (Supplemental Figure 1), it is apparent why many would assume that this precursor is the source of the previously unassigned peak. Interestingly, the sharp peak at larger q values present in the recrystallized indium myristate is not present in the drop cast indium myristate, indicating greater ordering and decreased spacing between ligands in the former, which would be somewhat expected in a sample that was recrystallized rather than drop cast. This same ordering and corresponding peak sharpness are also observed in the stearate and octadecylphosphonate capped indium phosphide particles in Figure 3. This is consistent with the hypothesis that the stearate and octadecylphosphonate ligands are in a more crystalline phase on the surface of the quantum dots.

However, the ^{1}H NMR spectra (Supplemental Figure 2) of the solutions used to drop cast the quantum dots for measurements depicted in Figure 3a and d (stearate capped and oleylamine exchanged indium phosphide) indicates otherwise. The spectra show the methyl and vinyl peaks of the bound capping ligands. Unbound ligands correspond to sharp ^{1}H NMR peaks upshifted from the broad peak attributed to ligands bound to the nanocrystal.[3, 4] These measurements indicate little unbound precursor after significant washing steps, corresponding to approximately 5 free stearates per nanocrystal for the sample capped with stearate. Considering the low scattering factors of the organic ligands,[5] unbound precursor cannot be the source of the peak in the powder X-ray diffraction patterns.

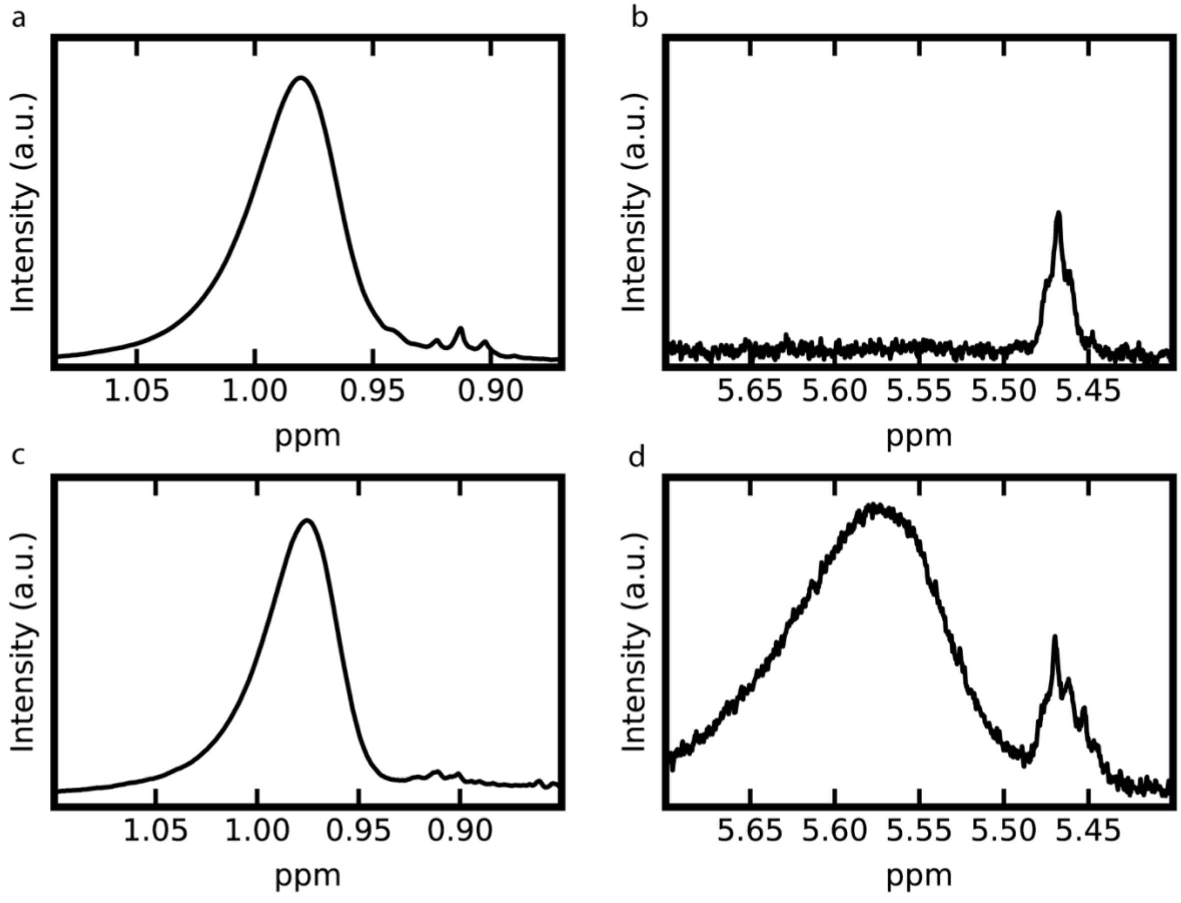

Supplemental Figure 2. ^{1}H NMR of ligand exchanges of indium phosphide quantum dots capped with stearate before and after exchange with oleylamine, taken in toluene-d_8. a,

Methyl peak showing little unbound ligand for native stearate capped indium phosphide quantum dots. **b**, Vinyl peak region showing an impurity in the ^{1}H NMR spectra. **c**, Methyl peak showing little unbound ligand for stearate capped indium phosphide quantum dots with 10% oleylamine exchanged. **d**, Vinyl peak region showing bound oleylamine peak.

The integrations of the areas beneath the peaks with the oleylamine exchange indicate that approximately 10% of the total ligand coverage is oleylamines, or bent ligands. This agrees nicely with the results by Balan et al., which indicate that the phase transition between the more ordered and less ordered phase of ligands should be below room temperature for a 10% bent ligand shell, while for 100% straight ligand shell this transition should occur above room temperature.[6]

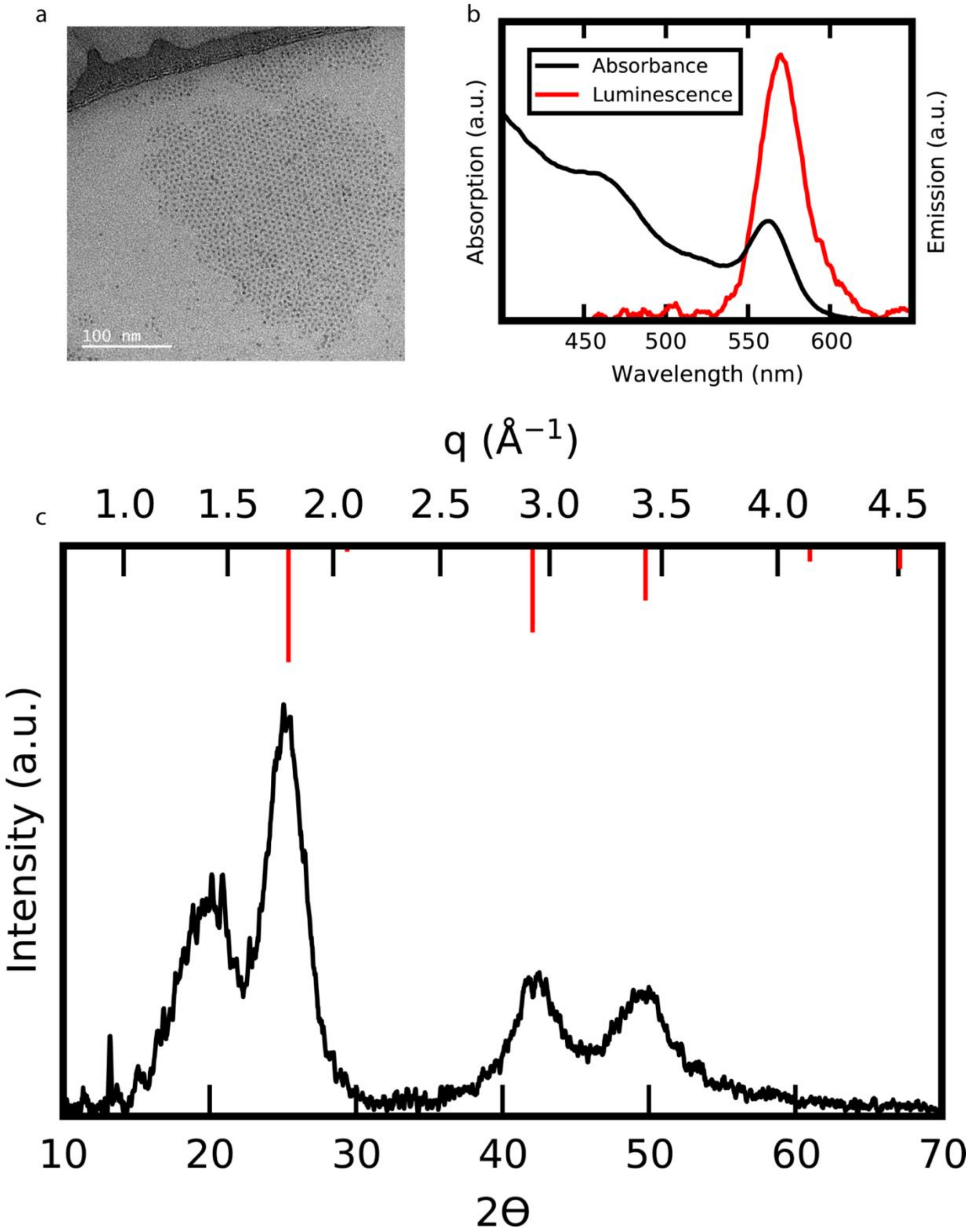
a
100 nm
b
Absorbance
Luminescence
Absorption (a.u.)
Emission (a.u.)
450 500 550 600
Wavelength (nm)
c
q (Å^{-1})
1.0 1.5 2.0 2.5 3.0 3.5 4.0 4.5
Intensity (a.u.)
10 20 30 40 50 60 70
2Θ

Supplemental Figure 3. Characterization of small cadmium selenide quantum dots. a, TEM image of cadmium selenide quantum dots. **b**, Absorption and emission spectra of cadmium selenide quantum dots. **c**, Experimental powder X-ray diffraction pattern of cadmium selenide quantum dots with reference peaks. Cu K-α ($\lambda = 1.5418$ Å) is the X-ray source for the powder X-ray diffraction spectrum.

The observation of the previously unassigned peak has been observed in other systems besides indium phosphide. Supplemental Figure 3 shows a powder X-ray diffraction pattern of cadmium sulfide quantum dots (4.3 nm diameter) capped with oleates where the ligand peak also appears (ICSD 1604283).

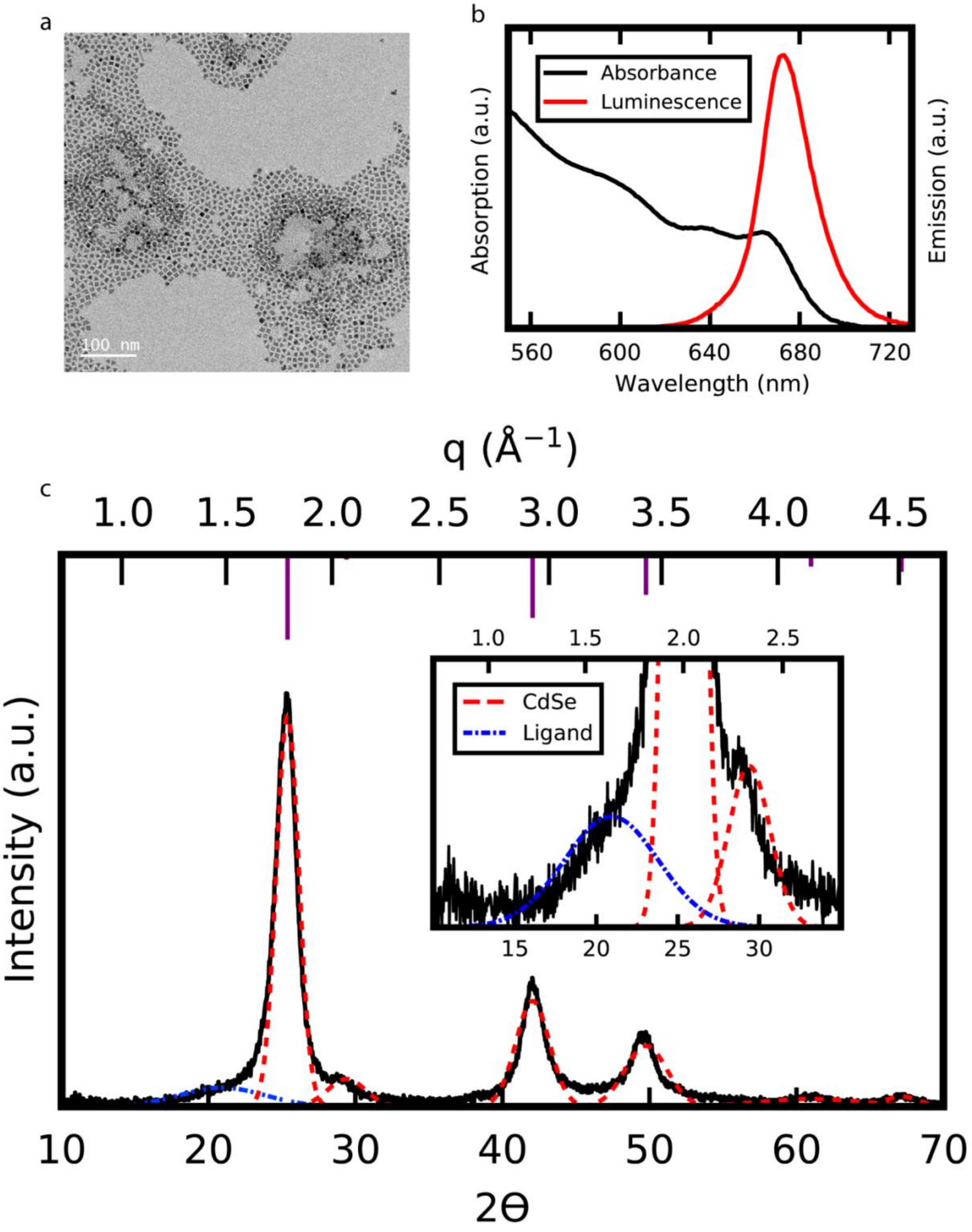

Supplemental Figure 4. Characterization of large cadmium selenide quantum dots. a, TEM image of cadmium selenide quantum dots showing approximately 10 nm diameter. **b**, Absorption

and emission spectra of cadmium selenide quantum dots. **c**, Experimental powder X-ray diffraction pattern of cadmium selenide quantum dots with reference peaks and peak deconvolution. Inset shows region where the oleate ligand powder X-ray diffraction peak appears. Cu K-α ($\lambda = 1.5418$ Å) is the X-ray source for the powder X-ray diffraction spectrum.

When particles become much larger, such as in the case of the cadmium selenide particles (approximately 10 nm diameter), the ligand peak becomes much less apparent when viewing the entire powder X-ray diffraction pattern. However, while diminished, the peak can still be deconvoluted with careful baseline subtraction, shown in Supplemental Figure 4 (ICSD 1611296).

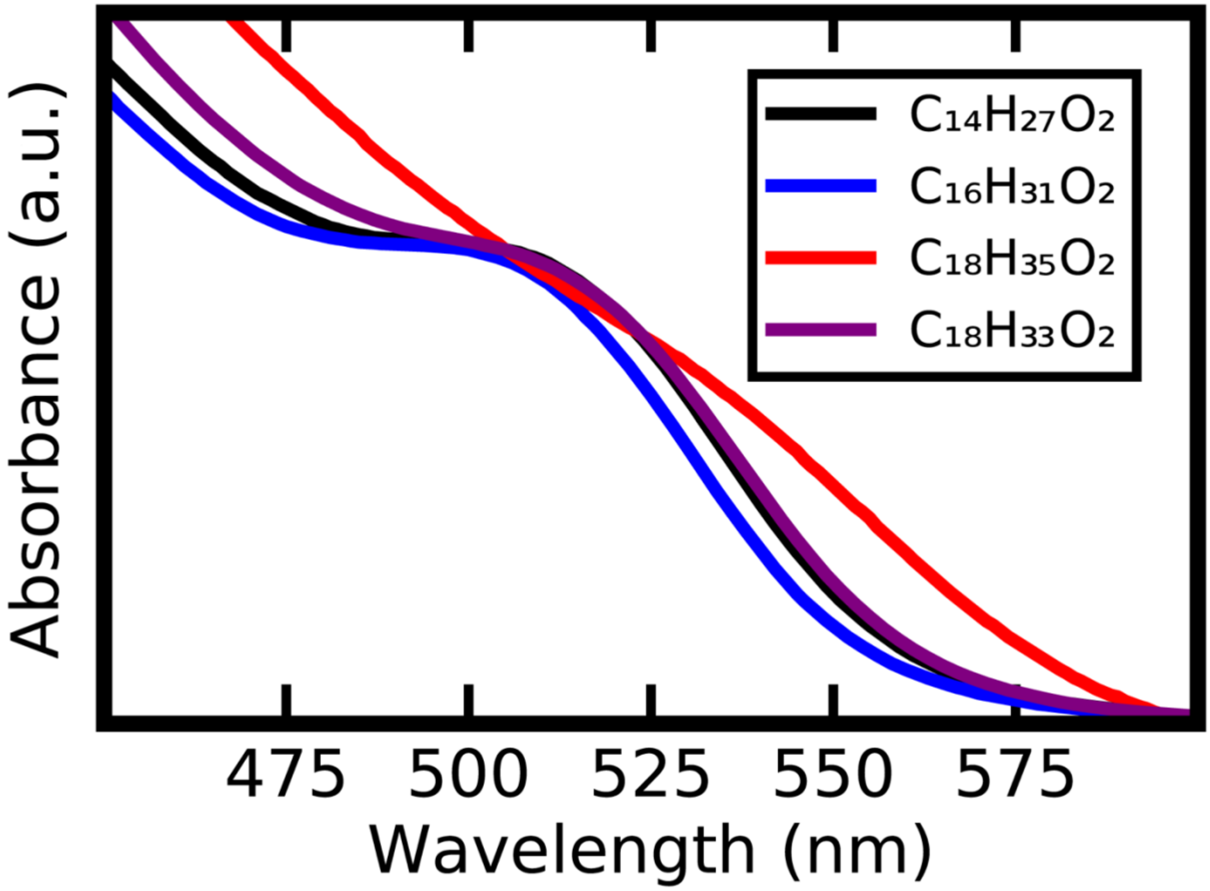

Supplemental Figure 5. Absorption spectra of indium phosphide quantum dots capped with myristate ($C_{14}H_{28}O_2$), palmitate ($C_{16}H_{32}O_2$), stearate ($C_{18}H_{26}O_2$), and oleate ($C_{18}H_{34}O_2$) ligands indicating size similarity.

The absorbance spectra of the indium phosphide quantum dots synthesized with different length capping ligands have previously been reported, but these measurements (Supplemental Figure 5) show that the QDs are similarly sized and thus isolating ligand length as the experimental variable in Figure 3 is reasonable.[3]

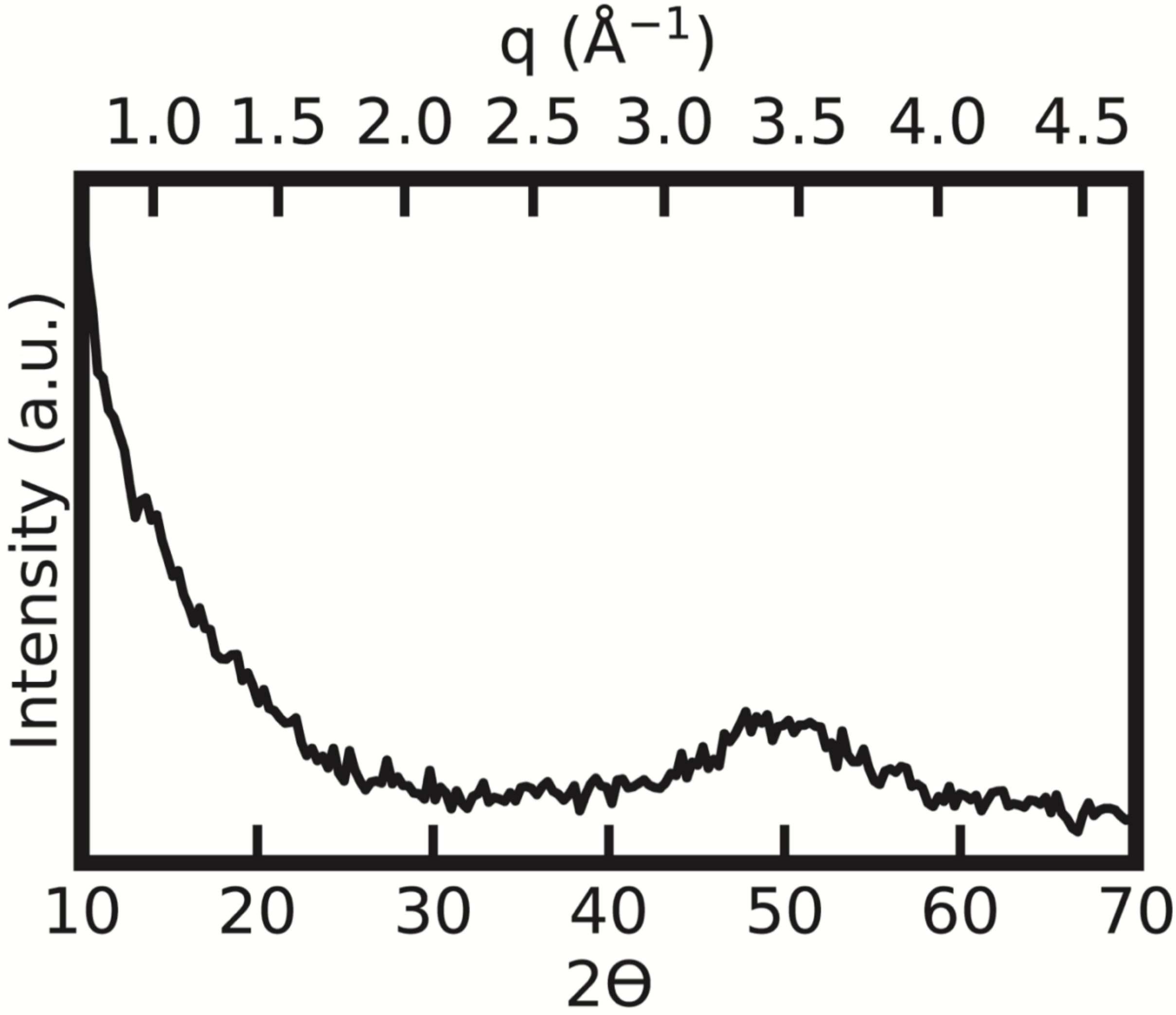

Supplemental Figure 6. Experimental powder X-ray diffraction background spectrum of silicon wafer. Cu K-α ($\lambda = 1.5418$ Å) is the X-ray source for the powder X-ray diffraction spectrum.

The previously unassigned peak also cannot be attributed to any background signal as it does not appear in the background powder X-ray diffraction pattern (Supplemental Figure 6).

Note on powder X-ray diffraction Instruments

The measurements in Figures 4E and F were taken on different instruments to optimize different variables. The instrument used for Figure 4E's measurements provides better signal to noise ratio allowing us to clearly see nuances in the ligand peak shape at different temperatures. Whereas, the diffractometer used for Figure 4F's measurements could accommodate a wider range of temperatures allowing us to observe ligand shell behavior at temperature extremes.

Supplementary Methods

Additional Chemicals: Cadmium acetate (99.995%), and selenium (99.99%), methyl acetate (99.5%), toluene-d_8 (99.8%), and mesitylene (98%) were purchased from Sigma Aldrich and stored in an argon glovebox.

Cadmium Selenide Quantum Dot Synthesis: Procedure was adapted from that reported by Liu et al. to form zinc-blende cadmium selenide nanocubes.[7] In a three-neck, oven-dried, 50 mL round-bottom flask 0.078 g of selenium (1.0 mmol) was combined with 15 mL of 1-octadecene with the inclusion of a Teflon-coated stir bar. After attaching a thermocouple adapter, septa cap, and air condenser column to the flask and greasing all glass joints with Apiezon H grease, the flask was attached to a Schlenk line, and evacuated at room temperature for one hour, reaching a pressure below 60 mTorr. The solution was the placed under argon and heated to 280 °C and maintained at this temperature for 30 minutes, transitioning to a clear, yellow solution. In tandem, in a three-neck, oven-dried, 50 mL round-bottom flask, 0.266 g of cadmium acetate dihydrate (1.00 mmol) was combined with 5 mL of technical grade oleic acid with the inclusion of a Teflon-coated stir bar. After attaching a thermocouple adapter, septa cap, and air condenser column to the flask and greasing all glass joints with Apiezon H grease, the flask was attached to a Schlenk line, and evacuated at room temperature for 15 minutes, reaching a pressure below 60 mTorr. The

solutions were heated under argon to 150 °C and then quickly drawn into a syringe and injected into the selenium flask, forming a red solution. The solution was then stirred at 265 °C for 2 minutes to form the small cadmium selenide quantum dots and 40 minutes to form the large cadmium selenide quantum dots before being rapidly cooled with a stream of hexanes and nitrogen, resulting in a black solution. The solution was cannulated under argon and transferred to an argon glovebox. In centrifuge tubes, 60 mL of methyl acetate was added to precipitate the particles. The solution was centrifuged at 5702 RCF for 5 minutes, forming a pellet. The supernatant was discarded, and the particles were resuspended in 8 mL of hexanes. An additional 24 mL of methyl acetate was added, and the solution was centrifuged at 5702 RCF for 5 minutes. After redispersing in 8 mL of hexanes, an additional 12 mL of methyl acetate was added followed by centrifuging at 5702 RCF for 5 minutes and the pellet was redispersed in 8 mL of hexanes.

Indium Myristate Synthesis: The synthesis of indium myristate used has previously been reported and characterized by FTIR, CHNS elemental analysis, and ^{1}H NMR.[4] For synthesis, 1.000 gram (3.425 mmol) of indium acetate was combined with 2.347 grams (10.28 mmol) of myristate acid alongside 20 mL of 1-octadecene in a three-neck, oven-dried, 50 mL round-bottom flask with a Teflon coated stir bar. After a thermocouple adapted, septa cap, and air condenser column was attached to the flask and all glass joints were sealed with Apiezon H grease, the flask was attached to a Schlenk line and heated to 110 °C under vacuum for one hour, leaving a clear solution. The flask was cooled to room temperature, forming a white precipitate. The entire flask was sealed under vacuum and transferred to an argon glovebox. The solution was poured into a centrifuge tube and centrifuged at 5702 RCF for 5 minutes. The supernatant was removed, and 20 mL of acetone was added to wash the solid with vigorous shaking. The solution was

centrifuged again, the supernatant discarded, and washed again with 20 mL of acetone. This process was repeated three times before the solid was added to a glass vial with 20 mL of acetone. After capping, the solution was heated on a hot plate until all of the precipitate dissolved. The solution was allowed to slowly cool to room temperature, and the indium myristate precipitated from solution. The solution was centrifuged one last time, the supernatant was discarded, and the precipitate was dried under a vacuum line overnight to remove residual acetone.

^{1}H NMR: ^{1}H NMR of indium phosphide quantum dots synthesized without hydrogen capped with stearate, palmitate, and myristate has previously been reported in tetrahydrofuran-d_8.[3] In an argon glovebox, samples were evaporated from the stock solution and redispersed in toluene-d_8. Internal mesitylene standard was 1% by volume. Measurements were performed on a Bruker Avance 700 instrument in oven-dried NMR tubes. Chemical shifts were referenced to the residual toluene signal. For quantitative measurements, the 90° pulse was calibrated, and samples were allowed to relax for 20 seconds between pulses. Uncertainty in the integration of NMR peaks was estimated at 5%.

Transmission Electron Microscopy: TEM imaging was performed on a FEI Tecnai T20 S-TWIN TEM operating at 200 kV with a LaB_6 filament using holey carbon TEM grids.

Additional Optical Characterization: Luminescence measurements were taken on a Horiba Jobin Yvon TRIAX 320 Fluorolog. Excitation wavelength was 437 nm with excitation and emission slit widths of 2.5 nm. The resolution was 1.0 nm, and each wavelength was integrated for 2 seconds.

Supplemental Table 1. List of sample references where this peak is observed in powder X-ray diffraction along with the explanation given (if any) and the material system.

Reason	Material	Reference
Organic material	CdSe, InP, PbSe	1, 2, 8, 9, 10, 11, 12
Forbidden reflection	CdSe	13, 14, 15
Substrate	CdSe, HgSe	16, 17
Inorganic impurity	InP, ZnCuInS, ZnS	18, 19, 20, 21, 22
No reason given	Ag_2S, CdS, CdSe, CdTe, PbSe, InAs, InP, ZnS	23, 24, 25, 26, 27, 28, 29, 30, 31, 32, 33, 34, 35, 36, 37

References

1. Lucey, D. W., *et al.* Monodispersed InP quantum dots prepared by colloidal chemistry in a noncoordinating solvent. *Chem. Mater.* **17**, 3754–3762 (2005).

2. Friedfeld, M. R., Johnson D. A., Cossairt B. M. Conversion of InP clusters to quantum dots. *lnorg. Chem.* **58**, 803–810 (2018).

3. Calvin, J. J., O'Brien E. A., Sedlak A. B., Balan A. D., Alivisatos A. P. Thermodynamics of composition dependent ligand exchange on the surfaces of colloidal indium phosphide quantum dots. *ACS Nano* **15**, 1407–1420 (2021).

4. Calvin, J. J., *et al.* Thermodynamic investigation of increased luminescence in indium phosphide quantum dots by treatment with metal halide salts. *J. Am. Chem. Soc.* **142**, 18897–18906 (2020).

5. Cromer, D. T., Mann J. B. X-ray scattering factors computed from numerical hartree–fock wave functions. *Acta Crystallogr. Sec. A* **24**, 321–324 (1968).

6. Balan, A. D., *et al.* Unsaturated ligands seed an order to disorder transition in mixed ligand shells of CdSe/CdS quantum dots. *ACS Nano* **13**, 13784–13796 (2019).

7. Liu, L., *et al.* Shape control of CdSe nanocrystals with zinc blende structure. *J. Am. Chem. Soc.* **131**, 16423-16429 (2009).

8. Lokteva, I., *et al.* Surface treatment of CdSe nanoparticles for application in hybrid solar cells: The effect of multiple ligand exchange with pyridine. *J. Phys. Chem. C* **114**, 12784-12791 (2010).

9. Mohapatra, P., Dung M. X., Choi J.-K., Jeong S.-H., Jeong H.-D. Effects of curing temperature on the optical and charge trap properties of InP quantum dot thin films. *Bull. Korean Chem. Soc.* **32**, 263-272 (2011).

10. Sahoo, Y., Poddar P., Srikanth H., Lucey D., Prasad P. Chemically fabricated magnetic quantum dots of InP: Mn. *The Journal of Physical Chemistry B* **109**, 15221-15225 (2005).

11. Wegner, K. D., Pouget S., Ling W. L., Carrière M., Reiss P. Gallium–a versatile element for tuning the photoluminescence properties of InP quantum dots. *Chem. Commun.* **55**, 1663-1666 (2019).

12. Du, H., *et al.* Optical properties of colloidal PbSe nanocrystals. *Nano Lett.* **2**, 1321-1324 (2002).

13. Antanovich, A., *et al.* A strain-induced exciton transition energy shift in CdSe nanoplatelets: The impact of an organic ligand shell. *Nanoscale* **9**, 18042–18053 (2017).

14. Chen, D., Gao Y., Chen Y., Ren Y., Peng X. Structure identification of two-dimensional colloidal semiconductor nanocrystals with atomic flat basal planes. *Nano Lett.* **15**, 4477-4482 (2015).

15. Li, Z., Peng X. Size/shape-controlled synthesis of colloidal CdSe quantum disks: Ligand and temperature effects. *J. Am. Chem. Soc.* **133**, 6578-6586 (2011).

16. McElroy, N., *et al.* Comparison of solar cells sensitised by CdTe/CdSe and CdSe/CdTe core/shell colloidal quantum dots with and without a CdS outer layer. *Thin Solid Films* **560**, 65-70 (2014).

17. Mirzai, H., *et al.* The room temperature phosphine-free synthesis of near-infrared emitting hgse quantum dots. *J. Mater. Chem. C* **2**, 2107-2111 (2014).

18. Granada-Ramirez, D., *et al.* Effect of the indium myristate precursor concentration on the structural, optical, chemical surface, and electronic properties of InP quantum dots passivated with zns. *J. Mater. Sci.: Mater. Electron.* **30**, 4885–4894 (2019).

19. Shweky, I., *et al.* Seeded growth of InP and InAs quantum rods using indium acetate and myristic acid. *Materials Science and Engineering: C* **26**, 788-794 (2006).

20. Lee, D., *et al.* Synthesis of InP nanocrystals using triphenyl phosphite as phosphorus source. *Korean J. Chem. Eng.* **36**, 1518-1526 (2019).

21. Wang, X., *et al.* A high efficient photoluminescence Zn–Cu–In–S/ZnS quantum dots with long lifetime. *J. Alloys Compd.* **640**, 134–140 (2015).

22. Hansen, E. C., Bertram S. N., Yoo J. J., Bawendi M. G. Zinc thiolate enables bright Cu-deficient Cu-In-S/ZnS quantum dots. *Small* **15**, 1901462 (2019).

23. Zhuang, Z., Peng Q., Wang X., Li Y. Tetrahedral colloidal crystals of Ag_2S nanocrystals. *Angew. Chem. Int. Ed.* **46**, 8174-8177 (2007).

24. Pan, D., Jiang S., An L., Jiang B. Controllable synthesis of highly luminescent and monodisperse CdS nanocrystals by a two-phase approach under mild conditions. *Adv. Mater.* **16**, 982-985 (2004).

25. Katsaba, A., *et al.* Surface states effect on photoluminescence of CdS colloidal nanocrystals. *J. Appl. Phys.* **113**, 184306 (2013).

26. Murray, C., Norris D. J., Bawendi M. G. Synthesis and characterization of nearly monodisperse CdE (E= sulfur, selenium, tellurium) semiconductor nanocrystallites. *J. Am. Chem. Soc.* **115**, 8706-8715 (1993).

27. Sokolikova, M., Vasiliev R., Gaskov A. Synthesis of quasi-two-dimensional colloidal cadmium selenide nanoparticles and formation of sulfide monolayer on their surfaces. *Russian Journal of Inorganic Chemistry* **59**, 413-418 (2014).

28. Lim, S. J., Chon B., Joo T., Shin S. K. Synthesis and characterization of zinc-blende CdSe-based core/shell nanocrystals and their luminescence in water. *J. Phys. Chem. C* **112**, 1744-1747 (2008).

29. Capitani, C., *et al.* Quantized electronic doping towards atomically controlled "charge-engineered" semiconductor nanocrystals. *Nano Lett.* **19**, 1307-1317 (2019).

30. Vasiliev, R. B., Dorofeev S. G., Dirin D. N., Belov D. A., Kuznetsova T. A. Synthesis and optical properties of PbSe and CdSe colloidal quantum dots capped with oleic acid. *Mendeleev Commun.* **14**, 169-171 (2004).

31. Tamang, S., Lee S., Choi H., Jeong S. Tuning size and size distribution of colloidal InAs nanocrystals via continuous supply of prenucleation clusters on nanocrystal seeds. *Chem. Mater.* **28**, 8119-8122 (2016).

32. Wegner, K. D., *et al.* Influence of the core/shell structure of indium phosphide based quantum dots on their photostability and cytotoxicity. *Frontiers in chemistry* **7**, 466 (2019).

33. Ramasamy, P., Ko K.-J., Kang J.-W., Lee J.-S. Two-step "seed-mediated" synthetic approach to colloidal indium phosphide quantum dots with high-purity photo-and electroluminescence. *Chem. Mater.* **30**, 3643-3647 (2018).

34. Liu, Z., *et al.* Coreduction colloidal synthesis of III–V nanocrystals: The case of InP. *Angew. Chem. Int. Ed.* **47**, 3540-3542 (2008).

35. Battaglia, D., Peng X. Formation of high quality InP and InAs nanocrystals in a noncoordinating solvent. *Nano Lett.* **2**, 1027-1030 (2002).

36. Kim, Y., *et al.* Bright and uniform green light emitting InP/ZnSe/ZnS quantum dots for wide color gamut displays. *ACS Appl. Nano Mater.* **2**, 1496-1504 (2019).

37. Wang, Y., *et al.* Raman scattering study of molecules adsorbed on ZnS nanocrystals. *J. Raman Spectrosc.* **38**, 34-38 (2007).
